# Using evidence briefs to support future implementation planning: the case of early fibrinolysis for myocardial infarction and rehabilitation for the amputee patient in Colombia

**DOI:** 10.1186/s12961-026-01466-5

**Published:** 2026-09-10

**Authors:** Marcela Vélez, Viviana Vélez-Marín, Luz Helena Lugo-Agudelo, Daniel Felipe Patiño Lugo, Pamela Velásquez-Salazar, Luisa Fernanda Mesa Franco, Claudia Yaneth Vera-Giraldo, Juan Carlos Velásquez-Correa, Christine Fahim, Robert Marten, Sonam Yangchen, Sharon Straus

**Affiliations:** 1 Faculty of Medicine, University of Antioquia, Cra 51d #62-29, Medellín, Antioquia Colombia; 2 Knowledge Translation Program, Li Ka Shing Knowledge Institute, St. Michael’s Hospital, 30 Bond Street, Toronto, ON M5B 1W8 Canada; 3 Alliance for Health Policy and Systems Research, 20 Avenue Appia, 1211 Geneva, Switzerland

**Keywords:** Implementation, Clinical practice guidelines, Early fibrinolysis, Rehabilitation, Amputee

## Abstract

**Background:**

Despite the relevance of clinical practice guidelines for health policy development, planning, evaluation and quality improvement, implementation of the Colombian national guidelines is still in early stages. The objective of this study was to develop two evidence briefs supporting future development of implementation plans.

**Methods:**

We involved Colombian Ministry of Health representatives in dialogues about prioritizing recommendations for implementation. Two groups of recommendations were selected, and we developed two evidence briefs using the SUPPORT methodology’s systematic process of searching and synthesizing evidence. One brief focused on providing early fibrinolysis when invasive therapy is not available promptly for patients with ST-elevation myocardial infarction (STEMI); and the second brief focused on providing rehabilitation services for individuals with amputations.

**Results:**

For the evidence brief on early fibrinolysis (EF) for individuals with a STEMI, 33 articles were included. Overall, 21 studies addressed barriers, 10 addressed facilitators, and 25 focused on strategies for implementation.

For the brief on rehabilitation services for persons with amputations, 45 articles were included. A total of 23 studies addressed strategies for adherence to policy recommendations in low- and middle-income countries. Moreover, 23 studies included comprehensive care and continuity of rehabilitation services for persons with amputations or patients currently experiencing or likely to experience a disability. In both briefs, we identified common facilitators and barriers for implementing recommendations. Common barriers related to patient characteristics include living in rural areas or low socioeconomic status communities. Other barriers include the lack of clinicians’ training to provide EF for individuals with a STEMI and rehabilitation services for individuals with amputations. A common implementation strategy was establishing networks integrating community facilities and hospitals, with clear healthcare pathways that facilitate navigating the health system and understanding the functions of each stakeholder.

**Conclusions:**

Both evidence briefs showed that in Colombia, to implement key recommendations, it is essential to improve timely access and continuity of services for populations living in rural areas and low-income settings. Evidence suggests that establishing networks integrating community facilities and hospitals might positively and significantly impact health outcomes and healthcare experience for patients with STEMI and patients with amputations who need rehabilitation services. The education sector is essential in improving the knowledge and skills of patients and healthcare providers of both conditions.

## Background

Guideline development has increasingly focused on planning how to effectively implement recommendations in the health system [1, 2]. This planning includes developing mechanisms and strategies that help identify, from a large set of evidence-based recommendations, the ones prioritized for implementation [1, 2]. The investment and effort that professional organizations, researchers and policy-makers place in developing guidelines should also extend to the process of implementation.

Between 2008 and 2016, the Colombian Ministry of Health (CMoH) funded the development of 58 national clinical practice guidelines (CPGs). Those guidelines aimed to reduce unexplained variations in medical care and improve the efficient management of limited resources [3]. The CPGs were developed using high methodological standards. Their development mobilized economic resources and the research capacity of many different institutions in the country (e.g. universities, hospitals, professionals and patient organizations). Despite the relevance of the CPGs for informing health policy development, planning, evaluation and quality improvement [4–6], implementation of CPG recommendations is still in the early stages. The overwhelming number of recommendations derived from CPGs in Colombia (*n* = 3887), lack of stakeholders’ experience in implementation and various implementation barriers [7–9] have left a large proportion of the recommendations unimplemented.

One strategy that may facilitate the implementation of recommendations is to engage stakeholders and policy-makers to prioritize recommendations on the basis of the strength of the evidence, people and health conditions addressed, and the feasibility of the implementation. Previously, we developed a tool that provides this kind of information to Colombian stakeholders and policy-makers [10]. We used this tool and the information contained to inform several dialogues with representatives of the CMoH. After this dialogue process, we prioritized two groups of recommendations for developing evidence briefs that support the implementation planning. The criteria used to select recommendations were based on the potentially significant impact on health outcomes, health professionals’ experiences and the use of health system resources. The first group of recommendations regards the management of ST-elevation myocardial infarction (STEMI) with the provision of early fibrinolysis (EF) when invasive therapy is not available on time [11, 12]. The second group involved recommendations about providing rehabilitation services for persons with amputations [13].

Early fibrinolysis is the option for coronary artery reperfusion in patients with STEMI who cannot access a primary percutaneous coronary intervention (PPCI) in less than 12 h from symptom onset or who cannot access medical assistance within 90 min [12, 14–17]. EF reduces the risk of death and reinfarction by up to 20% and reduces mid- and long-term morbidity and mortality [12, 14, 16–21]. This intervention might be the best option of coronary reperfusion therapy for most individuals with a STEMI in Colombia, given the low availability of PPCI, barriers to providing coronary healthcare services, and geographic and topographic conditions [14, 15, 18, 22–26]. There are no studies in Colombia reporting the number of persons with STEMI without access to EF, but between 2011 and 2019, 288 169 deaths were identified due to myocardial infarction [12].

Recommendations about rehabilitation services for persons with amputations were selected because of the importance of improving healthcare delivery for people with disabilities in the Colombian health system. Armed conflict contributes to the high number of amputees in Colombia; from 1990 to July 2021, antipersonnel mines have been responsible for amputating the limbs of 8451 people [27]. The amputation of a limb is a condition that constrains the functioning and participation of the affected persons; therefore, rehabilitation is an essential intervention to restore dignity and functioning to these patients. In two different reports, the World Health Organization and the United Nations requested that member countries strengthen and expand rehabilitation services for people with rehabilitation needs [28–30].

This study is part of extensive research that aims to promote evidence-informed decision-making about implementing health recommendations at the system level. The objective of this study was to develop evidence briefs that support the future development of implementation plans for (1) EF in persons with a STEMI when invasive therapy is not available promptly and (2) provision of rehabilitation services for persons with amputations.

## Methods

Two evidence briefs were developed by systematically searching and synthesizing evidence according to the SUPPORT methodology [31].

### Stakeholder involvement

Three resources were used for prioritizing recommendations in this study. First, we reviewed the information about recommendations pending for implementation contained in a tool developed by us [10] (pending publication in this same journal); second, we considered the two suggestions of the CMoH representatives; and third, we considered the conclusions of a stakeholder dialogue about comprehensive healthcare pathways for persons with amputations. The research team selected recommendations about providing early fibrinolysis in individuals with a STEMI when invasive therapy is not available promptly and rehabilitation services for individuals with amputations.

When the focus of the evidence briefs was decided, we contacted and met the acute coronary syndrome (ACS) and amputee guideline development leaders. In those meetings, we discussed their opinions and insights about the relevance and validity of recommendations prioritized, possible benefits and risks of their implementation, and possible barriers and facilitators to the implementation process.

We engaged representatives of the CMoH for the prioritization of recommendations for implementation. We conducted two 1-h virtual meetings with the director and the pharmaceuticals and health technologies division team at the CMoH to discuss which recommendations were prioritized for implementation. The representatives of the CMoH suggested (1) focusing on recommendations that might have, in the short term, a positive impact on health population outcomes and (2) prioritizing healthcare for persons with amputations in such a way that we use the insights and progress made in a stakeholder-deliberative dialogue about comprehensive healthcare pathways for persons with amputations [32].

### Search strategy

For both evidence briefs, searches were performed in PubMed and Health Systems Evidence. For the brief on EF for persons with a STEMI, we used variations of keywords such as myocardial infarction, thrombolytic therapy, implementation science and guideline adherence in five additional databases (i.e. Embase, Cochrane, Lilacs, Bireme and SciELO). For the brief on rehabilitation, we used variations of keyword such as amputation, disability, developing countries, implementation science and guidelines (search strategies are provided in Additional File 1). We carried out a grey literature search for each evidence brief to identify ongoing research, key articles suggested by experts, documents of ministries of health in different countries and recommendations from expert groups and international organizations about barriers, facilitators and strategies for implementation. All the searches were performed between February and May 2021 by an experienced documentalist, who developed the search strategies through an iterative process in consultation with the research team.

### Inclusion assessment

For each evidence brief, two independent reviewers performed the inclusion assessment of titles and abstracts after a calibration exercise with 10 randomly selected articles. Conflicts were solved by consensus or, in some cases, by a third researcher. The full-text inclusion assessment was performed by one researcher in each brief (P.V. and V.V.).

For the brief on EF for persons with a STEMI, we included studies that (1) focused on persons with a STEMI 18 years or older and (2) described implementation strategies providing early fibrinolysis when invasive therapy is not available promptly. Regarding the brief on rehabilitation services for persons with amputations, we included studies that (1) focused on persons with amputations currently experiencing a disability or who are likely to experience disability and (2) described implementation strategies for providing comprehensive healthcare and continuity of rehabilitation services in low- and middle-income countries. The third criterion in both briefs was including studies reporting impact in the quadruple aim (i.e. health outcomes, patient experiences, costs for the health system and provider experiences), as well as barriers and facilitators. Studies were not excluded on the basis of their design, quality or publication language.

### Data extraction and quality assessment

For both evidence briefs, one researcher performed data extraction. Three researchers participated in data extraction for the brief on EF for individuals with a STEMI (V.V., C.V. and M.V.), and four researchers on rehabilitation services for persons with amputations (P.V.S., L.F.M., J.S.F. and D.P.). For each brief, we extracted bibliographic information, study characteristics, outcomes assessed, implementation strategies addressed, health outcomes, patient experiences, costs for the health system, provider experiences, barriers and facilitators.

One researcher performed a quality assessment in each brief (P.V. and V.V.). We used quality appraisal tools appropriate to the type of study design: AMSTAR II for systematic reviews [33], AGREE GRS for CPGs [34] and the critical appraisal tools developed by the Joanna Briggs Institute (JBI) for other types of studies (e.g. quasi-experimental, cohort, cross-sectional, qualitative and other designs) [35]. All reviewers were trained at the beginning of the inclusion assessment and during the data extraction process.

### Data analysis

Data analysis proceeded in three stages. First, a single investigator summarized all the findings from the scientific articles included, which were mapped according to barriers, facilitators, strategies for implementation and impact in the quadruple aim (i.e. health outcomes, patient experiences, costs for the health system and provider experiences). We then included information from other non-peer-reviewed sources, such as policy documents, reports of international organizations and ongoing research. Third, to complete the categories of barriers, facilitators and strategies for implementation, we incorporated opinions, views and experiences of the relevant guideline development leaders and key issues discussed in the stakeholder dialogue about comprehensive healthcare pathways for persons with amputations.

The implementation strategies identified were organized into three categories for both briefs: (1) patients and caregivers, (2) healthcare providers and (3) health system. For the evidence brief on rehabilitation for persons with amputations, two elements were proposed: the first focused on strategies for adherence to policy recommendations in low- and middle-income countries, and the second focused on healthcare comprehensiveness and continuity of rehabilitation services for individuals with amputations.

## Results

### Evidence brief on early fibrinolysis for persons with a STEMI

#### Literature search

**Fig. 1:**
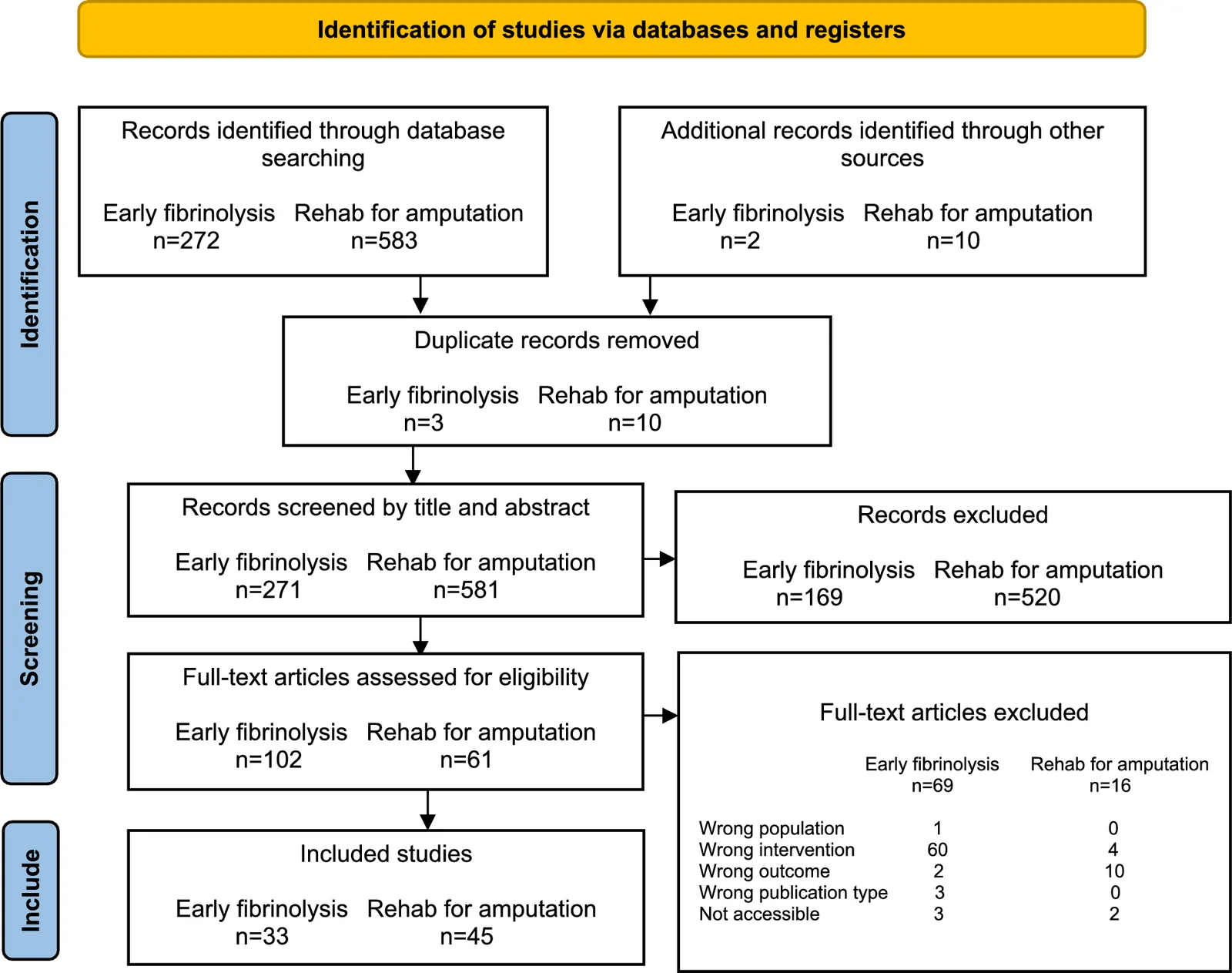
PRISMA flowchart

After duplicate removal, 271 titles and abstracts were identified and screened, and 33 studies were included in the brief: 6 CPGs or consensus [12, 16, 17, 19, 36, 37], 2 systematic reviews [38, 39], 2 quasi-experimental studies [40, 41], 3 qualitative studies [22, 23, 42], 11 observational studies [26, 43–52], a book chapter [53], and 8 nonsystematic reviews [14, 15, 18, 20, 24, 54–56] (see Fig. 1 for Preferred Reporting Items for Systematic Reviews and Meta-Analyses [PRISMA] chart).

#### Characteristics of studies included

Studies covered 48 countries from low-, middle- and high-income settings. Overall, 21 studies addressed barriers for implementation, 10 studies focused on a variety of facilitators and 25 studies referred to strategies for surpassing the barriers for implementation. Implementation plans were described in six studies, including a CPG, a quasi-experimental study, one book chapter and three nonsystematic reviews (see Additional File 2a for a summary of the characteristics of the studies included).

#### Quality assessment

Overall, the quality of the studies included was high for 21 studies: 4 CPGs [12, 16, 17, 36], 2 quasi-experimental studies [40, 41], 2 cohorts [47, 50], 1 cross-sectional [46], 3 qualitative studies [22, 23, 42], a book chapter [53], and 8 nonsystematic reviews [14, 15, 18, 20, 24, 54–56]. Nine studies were appraised as medium quality, which included two CPGs [19, 37], six cohorts [26, 43, 48, 49, 51, 52] and one case–control study [45]. Lastly, one cross-sectional study was appraised as low quality [44], and two systematic reviews as critically low [38, 39]. The summary of the quality appraisal for the articles included in this evidence brief is provided in Additional File 2a.

#### Main findings

**Fig. 2:**
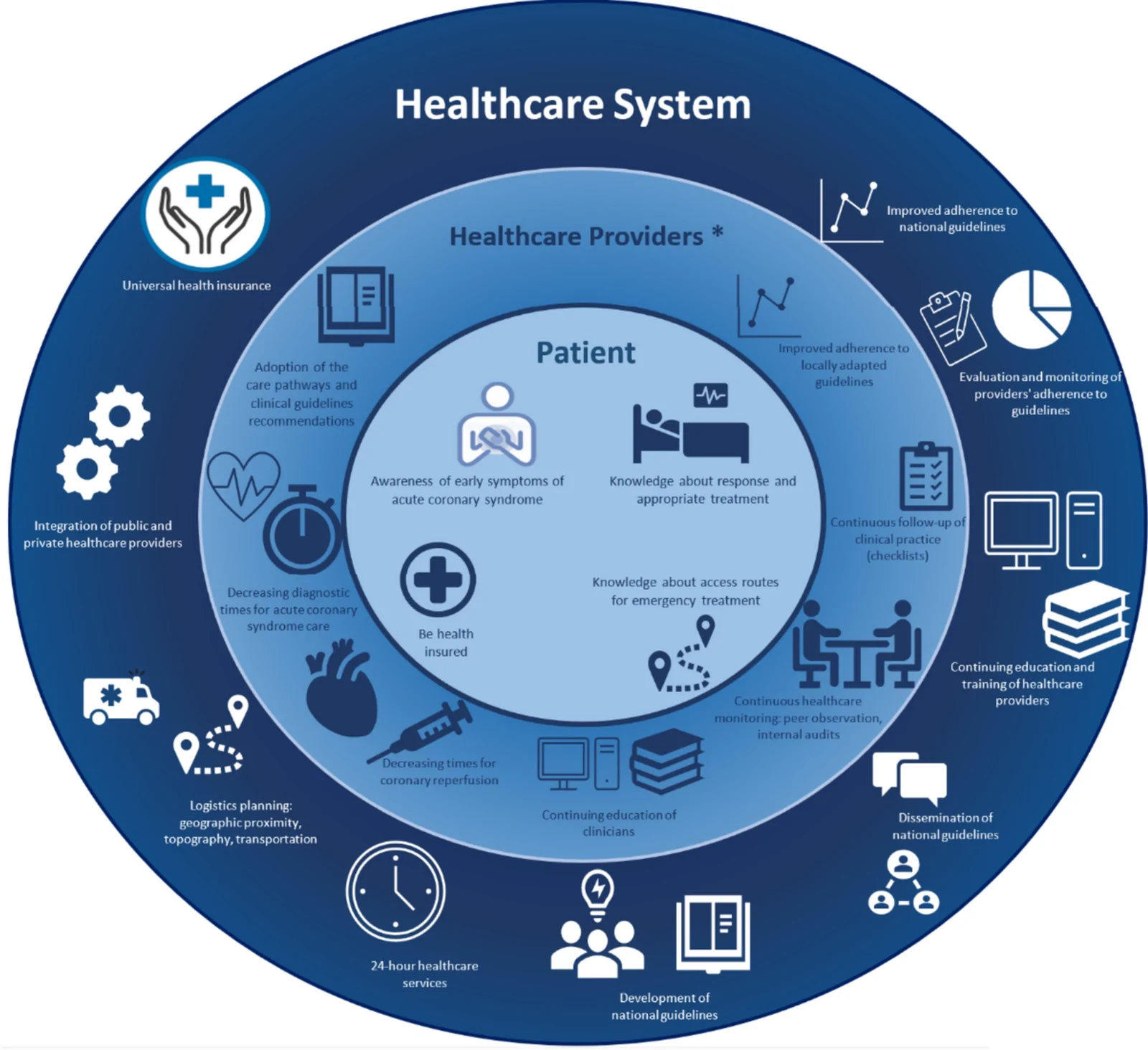
Facilitators and strategies for implementing early fibrinolysis in persons with a STEMI. Own creation based on the review of the evidence used for the development of the brief. ^*^Healthcare providers refers to health insurance managers, healthcare institutions, prehospital care providers or clinicians

Findings are presented in three subsections: (a) Barriers, (b) Facilitators and (c) Strategies for implementation. These findings were organized according to the actor involved in the process (i.e. patients, healthcare providers and health system) (Fig. 2).

#### Barriers

At the patient level, the principal barrier identified was the late consultation for medical attention due to lack of recognition of symptoms of acute myocardial infarction (AMI). This issue has been identified by multiple studies in different countries as responsible for two thirds of the delays for receiving myocardial reperfusion [14, 22, 24, 26, 44, 53, 54, 56]. Certain patient characteristics were associated with low accessibility to myocardial reperfusion (both pharmacological and mechanical), among them, a low socioeconomic status and low educational level [22, 24, 48, 53, 54, 56]; residing in rural areas and having difficulties in access to transportation [15, 16, 22, 44, 51, 53, 54, 56]; race [48, 53]; and lack of medical insurance [15, 22, 24, 49, 54, 56]. All the barriers identified are summarized in Table 1.

**Table 1:** Barriers and facilitators for implementing early fibrinolysis in persons with a STEMI

Level	Barriers	Facilitators
Patient	• Delayed recognition of ACS symptoms, including unusual presentations [14, 22, 24, 26, 44, 53, 54, 56] • Low socioeconomic status and educational level [22, 24, 48, 53, 54, 56] • Residence in rural and disperse areas [15, 16, 22, 44, 51, 53, 54, 56] • Difficulties in accessing transportation (public and ambulances) owing to geographic conditions, affordability or lack of availability during the night [15, 22, 53, 54, 56] • Lack of medical insurance, including problems associated with the quality of the insurance coverage [15, 22, 53, 54, 56] • Racialized populations having poorer access to healthcare [48, 53]	• Existing educational campaigns for primary and secondary prevention for ACS, expanding its coverage to include education on ACS symptoms and cardiovascular risk factors, and access routes to the health system [22, 42, 49] • Dissemination of ACS guidelines among patients [22, 49, 53] • High percentage of insured population in Colombia (98.57%) [22, 53]
Healthcare providers Individual level	• Colombian CPG for the management of ACS is long and impractical to read [12, 14, 22, 23, 42, 53, 55] • Delays in recognition and diagnosis of STEMI by the first responding healthcare provider. This includes difficulties for interpreting the electrocardiogram (ECG) or lack of availability of it [16, 20, 22, 24, 26, 42, 44, 47, 53, 56] • Inadequate triage of patients with chest pain (diagnosis and treatment delays) [22, 23, 26, 42, 53] • Lack of confidence of the healthcare provider in administering EF, either in primary care or prehospital settings. This was associated with a lack of training and experience that affects the confidence of health personnel; the belief that these procedures are not part of the scope of practice of general physicians or paramedics; the fear concerning referral delay to a specialized centre; and the perception of increased risk of harm when providing fibrinolysis in the community context [16, 20, 22, 24, 26, 42, 44, 47, 53, 56]	• Healthcare providers’ desire to have training in EF, including in prehospital settings [22, 42, 44] • Disposition of healthcare providers to accept new interventions for the improvement of the attention for these patients [42, 44, 53]
Healthcare providers Institutional level	• Difficulties for patients’ referral and transfer, wrong transportation decisions, lack of ambulances and absence of knowledge about health system functioning; geographical and environmental factors associated with traffic, road conditions, climate and the distance of routes from remote areas, among others, also had an influence [18, 22–24, 26, 36, 37, 44, 46, 47, 53, 55] • Administrative delays related to access to medical records, confirmation of the patient’s insurance, lack of coordination between prehospital and emergency services, and procedures of medical codification and billing [18, 22, 24, 47, 53, 55] • Lack of standardized, locally adapted guidelines for STEMI care [12, 14, 22, 23, 42, 53, 55] • Delays in timely access to in-hospital reperfusion, associated with fibrinolytic availability, the hour at which patients require medical attention, concerns for the safety in the administration of the medications, and lack of training and experience in administering the medications [18, 22–24, 37, 42, 45, 53, 55] • The underlying deficient infrastructure in low-income settings can present overwhelming challenges to reorganize health delivery systems services owing to situations such as the lack of specialized staff trained in ACS management, the lack of resources, and the disposition of the administrative staff to have fibrinolytic medications available [18, 22–24, 37, 42, 45, 53, 55] • Lack of internal audit and quality control of each healthcare provider [18, 22, 24, 42, 45, 53, 54]	• The national good quality guidelines that are already available could be tools to structure locally appropriate recommendations that take into account the regional context, with information that is easy to consult and understand for any healthcare provider [22, 49, 53] • The national guidelines recommend EF as one of the main treatments to diminish morbidity and mortality associated with STEMI [12]
Health system	• Increased number of patients with lifestyle-related diseases, with the burden upon the countries’ health system passing through the epidemiological transition process [22, 53] • Lack of priority setting for investment on prevention of noncommunicable disease [22, 53] • Lack of medical insurance or low quality of health coverage [15, 22, 24, 53–55] • Fragmented healthcare network [15, 19, 22, 24, 37, 44–47, 51, 53, 56] • Scarcity of resources for the care of persons with a STEMI; lack of ambulances for transportation between institutions; lack of trained staff to make the prehospital diagnosis of STEMI; shortage of facilities with percutaneous coronary intervention (PCI) capacity, specialized personnel and coronary care units; and absence of electrocardiographs in every hospital [18, 22, 37, 44–46, 51, 53, 55] • Ambiguous definition of emergency stabilization for STEMI, making fibrinolysis not considered an emergency treatment [22, 37, 53] • Lack of clinical governance [22, 37, 53] • Lack of research at the local level [22, 53, 56] • In Colombia, both tenecteplase and reteplase continue to not be included in the benefit plan, and these are expensive drugs [12, 55, 57]	• The distribution of the health system by care complexity levels can help build geographically organized CCNs. These networks are facilitators for EF within the countries that implemented them [16, 19] • The percentage of the population with health insurance (98.57%) directly impacts the management of STEMI [22, 53] • The clinical governance implemented so far in the country should be the initial step for better-performing surveillance systems approaching healthcare in persons with a STEMI [18, 22, 42, 44, 53] • The inclusion of fibrin-specific medications (alteplase) in the benefit plan of health by the CMoH, through resolution 2481 expedited on 24 December 2020 [57]

At the healthcare provider level, we identified barriers at the individual and institutional levels. At the individual level, the most relevant barrier was the difficulty in complying with the national guidelines given their length or lack of adaptation to the local context [12, 14, 22, 23, 42, 53, 55]. The delay in recognizing symptoms and starting treatment of individuals with a STEMI was identified as related to clinicians’ lack of training and confidence in their skills to interpret the ECG and administer the fibrinolytic therapy [16, 20, 22, 24, 26, 42, 44, 47, 53, 56]. At the institutional level, we identified barriers related to the process of patient referral [18, 22–24, 26, 36, 37, 44, 46, 47, 53, 55]; administrative delays associated with health insurance [18, 22, 24, 47, 53, 55]; scarcity of human resources, equipment and medications in institutions providing healthcare for patients with STEMI [18, 22–24, 37, 42, 45, 53, 55]; and lack of audit and feedback within institutions [18, 22, 24, 42, 45, 53, 54].

The most significant barriers identified at the health system level were the lack of priority setting and funding for noncommunicable diseases, mainly in countries in epidemiological transition facing the burden of transmissible and noncommunicable diseases [22, 53]. Other critical barriers identified were the lack of health insurance [15, 22, 24, 53–55], the segmentation of the health system [15, 19, 22, 24, 37, 44–47, 51, 53, 56], high costs associated with the use of fibrin-specific drugs in the management of persons with a STEMI [12, 55, 57] and an ambiguous definition of emergency care in the context of STEMI [22, 37, 53]. Other barriers were related to the scarcity of resources at the health system level (e.g. lack of coronary care unit beds, PCI centres and fibrinolytic medications) [18, 22, 37, 44–46, 51, 53, 55], lack of clinical governance that enhances the adherence to CPGs [22, 37, 53], poor local research in cardiovascular diseases [22, 53, 56] and an inefficient process of audit and feedback [18, 20, 22, 24, 44, 53, 54].

#### Facilitators

At the patient level, the principal facilitators were related to using the infrastructure and experience of cardiovascular disease preventive programs that already exist, focusing on the recognition of AMI symptoms [22, 42, 49] and disseminating plain language guidelines among patients [22, 49, 53]. The facilitators identified are summarized in Table 1 and shown in Fig. 2.

Facilitators at the individual healthcare provider level were related to the desire of healthcare staff to receive training on the diagnosis and management of EF in persons with a STEMI [22, 42, 44] and the disposition to accept new interventions for these patients [42, 44, 53]. At the institutional level, we identified that good quality national CPGs are a tool that facilitates the adaptation of recommendations to the local and regional context, principally when local guidelines are easy for any healthcare provider to consult and to understand [22, 49, 53]. Other facilitators at the institutional level were a process of quality monitoring between the healthcare institution and the prehospital provider, which helps to identify, on time, malfunctioning equipment (e.g. electrocardiogram equipment) or other sources of suboptimal healthcare [42, 44].

At the system level, regional coronary care networks (CCNs) are the main facilitator for EF delivery [16, 19]. Other important facilitators are the adequate funding of cardiovascular research and the investment in establishing health information systems that report reliable epidemiological data, allowing a process of audit and feedback [18, 22, 42, 44, 53]. Different countries also noted that one facilitator is instituting payment reimbursement methods, such as government-covered insurance specifically for STEMI care, ensuring healthcare coverage for these patients within the regional care networks [24, 56].

#### Strategies

The objectives of any implementation strategy involving EF in STEMI should be, first, in the prehospital settings, to promote the recognition of STEMI symptoms by patients and reduce delays in accessing reperfusion treatment, and second, aggressive and early reperfusion at the hospital level using an appropriate combination of medications [16, 19, 38, 41, 50, 53].

Regional and national CCNs with predetermined pathways for coronary reperfusion (i.e. integrating community centres and highly complex facilities) were the most widespread strategy used to improve STEMI management. These networks can be adapted according to the setting and should involve all important actors in the healthcare management of persons with a STEMI, supporting access to timely treatment, which reduces morbidity and mortality [15, 17, 18, 24, 36, 49, 50, 54, 55].

At the patient level, these strategies focused on education on recognizing ACS symptoms and providing information about proper healthcare pathways to consult [22, 24, 41, 49, 53]. Information about CCNs can be provided using mass messages through communication media, text messages and email sent by the healthcare providers [22, 49, 54]. Studies also mentioned that patients should be aware of and educated about treatment costs and the importance of insurance; this knowledge facilitates patients’ ability to comply with long-term treatment while being mindful of health system resources [16, 24].

At the individual healthcare provider level, facilitators included sharing the same care pathways and healthcare staff in the CCNs [16, 24, 41, 50]. The commitment to adhere to recommendations from locally appropriate guidelines is also a meaningful strategy at this level [22, 49, 50, 53]. At the institutional level, we identified strategies to encourage the implementation of locally relevant CPGs [15, 16, 22, 53, 54], which include training programs for prehospital and in-hospital healthcare staff [15, 22, 43, 53], and monitoring and feedback for improving the intra- and interinstitutional healthcare processes [15, 22, 41, 43, 53].

At the health system level, we identified two main strategies. The first one focused on developing education programs according to the community context, taking equity considerations into account (e.g. communities of low socioeconomic status, low educational levels, or residing in rural areas, or racialized communities) [15, 22, 38, 51, 53]. The second strategy was oriented to developing health policies that prioritize and facilitate the establishment of CCNs [14–16, 19, 36–38, 41, 43, 49, 50, 53–55], which included adopting an explicit definition for emergency stabilization in the STEMI context [15, 22, 24, 37, 38, 49, 53] and implementing strategies to surpass the challenges associated with funding the costly healthcare delivery for individuals with a STEMI [15, 49, 53, 54].

### Evidence brief on the provision of rehabilitation services for an individual with amputation

#### Literature search

After duplicate removal, 573 titles and abstracts were identified and screened, and 45 studies were ultimately included in this brief: 1 overview [58], 15 systematic reviews [59–73], 2 evidence summaries [74, 75], 5 scoping reviews [76–80], 18 nonsystematic reviews [81–98], 1 multiple case study [99], 2 realist syntheses [100, 101] and a policy research plan [102] (see Fig. 1 for the PRISMA chart).

#### Characteristics of studies included

For element one, which addressed strategies for adherence to policy recommendations in low- and middle-income countries, we included 1 overview [58], 4 systematic reviews [61, 62, 67, 71], 3 scoping reviews [76–78], 12 narrative reviews [82, 84–89, 91, 94, 96–98], a policy research plan [102] and 2 evidence briefs [74, 75]. For element two, regarding comprehensiveness and continuity of rehabilitation services for persons with amputations, we included 11 systematic reviews [59, 60, 63–66, 68–70, 72, 73], 3 scoping reviews [78–80], 6 narrative reviews [81, 83, 90, 92, 93, 95], 2 realist syntheses [100, 101] and 1 multiple case study [99] (see Additional File 2b for a summary of the characteristics of the studies included).

#### Quality assessment

The quality of the systematic reviews included was appraised as high in five studies [62, 65, 69, 71, 72], moderate in six [61, 63, 64, 68, 70, 73], low in two [60, 66] and critically low in two [59, 67]. Five scoping reviews were appraised as moderate quality [76–80]. For this brief, quality was only assessed in systematic and scoping reviews. The summary of the quality appraisal for the articles included in this evidence brief is provided in Additional File 2a.

#### Main findings

**Fig. 3:**
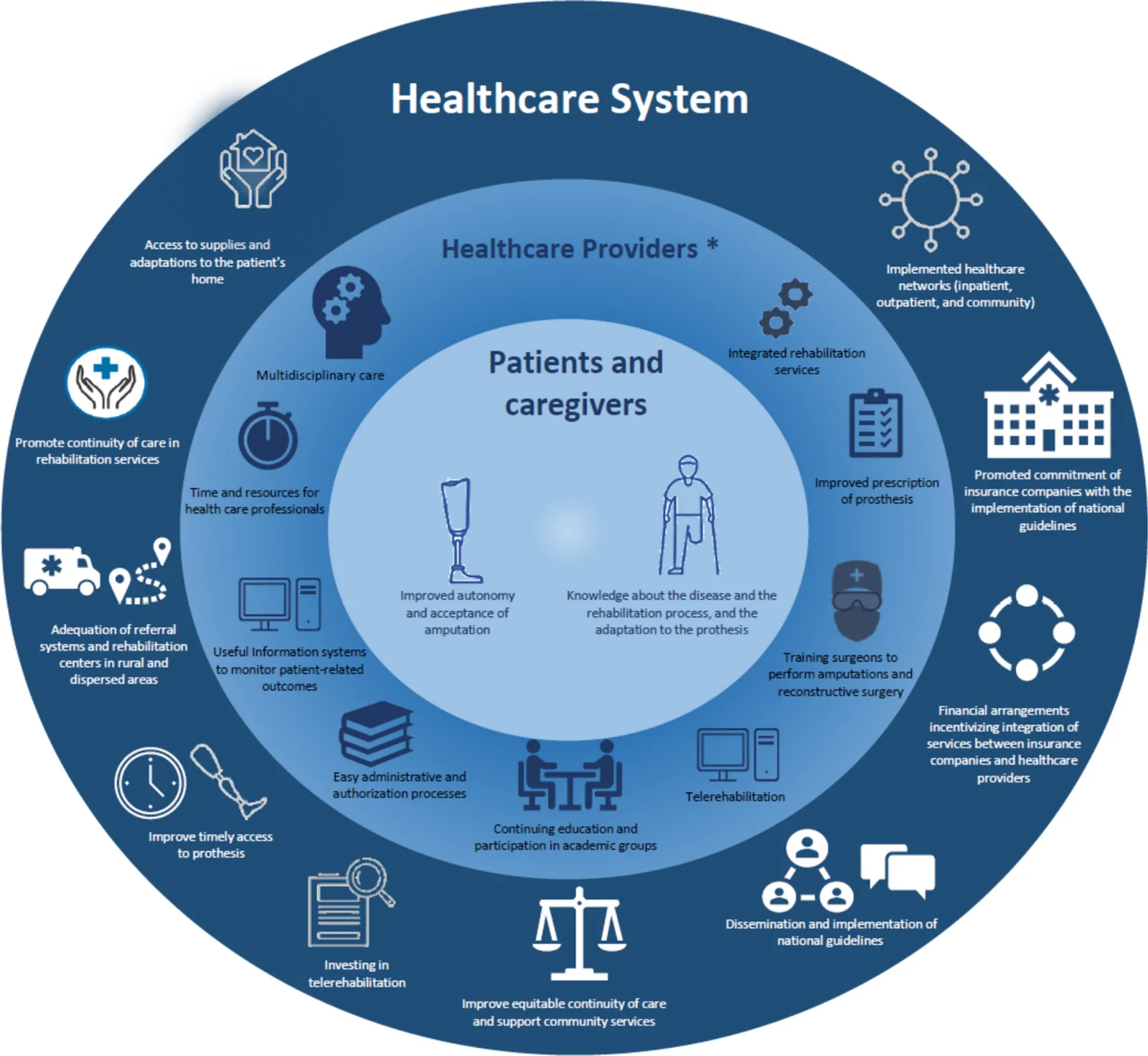
Facilitators and strategies for implementing early fibrinolysis in rehabilitation for persons with amputations. Own creation based on the review of the evidence used for the development of the brief. ^*^Healthcare providers refers to health insurance managers, healthcare institutions, prehospital care providers or clinicians

Findings are presented in three subsections: (a) Barriers and facilitators, (b) Strategies to improve adherence to policy recommendations in low- and middle-income countries and (c) Strategies for comprehensive and continuity of rehabilitation services for persons with amputations (Fig. 3).

#### Barriers and facilitators

Some patient characteristics were identified as barriers. Among these barriers are age (i.e. older people have more accessibility problems), residence in a dispersed geographic area with low access to healthcare services, insufficient knowledge of the condition, low acceptance of the amputation and lack of training in the use of prosthesis. The barriers identified are summarized in Table 2.

**Table 2:** Barriers to implementing rehabilitation services for persons with amputations [32, 108]

Level	Barriers
Patients and caregivers	• Age: Older age was related to barriers to implementing recommendations related to their participation in the amputation decision, rehabilitation process and prosthesis adaptation • Patient characteristics and the context in which patients live were related to barriers to the prescription of a prosthesis • Lack of knowledge of their disease in patients who require amputation owing to a medical cause and little or no participation of the patient and their family in making decisions about the amputation • Acceptance of the amputation and use of the prosthesis by the patients • Lack of autonomy, low self-esteem and communication difficulties in the implementation of recommendations related to the rehabilitation, recovery and adaptation of the patient to the prosthesis • Lack of training and qualification for the use of the prosthesis, lack of guarantees of affiliation to the healthcare system
Healthcare providers Individual level	• Perceived barriers regarding the amputee care provider related to their academic training, continuing education and participation in academic groups • Lack of academic training of health professionals in CPGs and evidence-based medicine, fear of following certain recommendations owing to legal consequences • Lack of personnel with adequate training to perform amputations and reconstructive surgery; this is most important at low-complexity institutions • Lack of personnel to ensure access and adaptation of the prosthesis • Delays in accessibility to the prosthesis owing to early prescription failures • Barriers given by the hiring of health personnel with fees for physical therapy, occupational therapy and psychology care. The care models in these circumstances with group care and different pathologies do not allow dedicating the time and resources necessary for patients with complex rehabilitation needs such as amputees • There are no mental healthcare programs integrated into the rehabilitation processes. Many health professionals are unaware of these patient needs and the support that must be given
Healthcare providers Institutional level	• Lack of integration in the comprehensive rehabilitation process since, in many cases, each intervention is carried out in a different place • Lack of integration networks between health institutions of different levels of complexity owing to fragmentation of care, more administrative procedures, health complications in patients and missed appointments • Lack of an integrated information system for scheduling appointments or sending appointment reminders • Barriers that arise from information systems and the measurement of indicators and outcomes in patients; underreporting and lack of a single information system • The type of financial arrangements between insurance companies and healthcare institutions promoting fragmentation and requiring a patient to attend different institutions to receive the services required • Lack of timely payments from insurance companies to healthcare institutions • Frequent regulatory changes in the system pose barriers to implementing CPGs in accordance with the content of the benefits plan • Barriers that arise owing to the lack of access to services and technologies: limited access to diagnostic aids, supplies and adaptations in the patient’s home that allow the adequate use of prostheses. In rural areas, there are no interdisciplinary teams or prosthetic manufacturing workshops • There are also difficulties in the authorization system used by insurers, with limitations in access for users and lack of agility in the authorization processes and administrative procedures • Barriers that arise owing to the limited availability of adequate infrastructure or the lack of availability or authorization of rehabilitation services in rural and dispersed areas • The site where the service is provided, the training of the providers and the performance evaluation are barriers to implementation
Clinical practice guideline	• Barriers due to the design of the CPG document, the characteristics of the developer group and the dissemination process • Barriers due to lack of knowledge to understand technical concepts within the guide, such as the levels of evidence and the strength of the recommendation • Lack of dissemination • Lack of credibility in local/institutional guidelines • The recommendation of expensive technologies that are not included in the benefits plan • The difficulty of inclusion of products and services recommended in the health benefits plan and their availability in different regions of the country • Barriers due to lack of participation of insurers in the preparation of CPGs and in their availability for the implementation of the recommendations and milestones defined in the Path for Integrated Healthcare (RIAS)
Health system	• Lack of integrated networks between healthcare institutions and fragmentation of administrative processes • Type of financial arrangements between insurance companies and healthcare institutions (i.e. insurance companies change the contracts with healthcare institutions, continuously forcing patients to move to different healthcare providers) • Amputees are not among the top 10 causes of consultation in a general rehabilitation service, and therefore, clinicians lack the knowledge and expertise for the management of this condition, and implementing the guideline is not a priority • For insurance companies, some of the components of the prostheses are not cost-effective • In low- and middle-income countries, leadership, governance and policy issues are major barriers • In these countries, in addition to the lack of resources, there is the weakness of the health systems, the organizational capacity and the management of existing resources

The most common barrier identified at the healthcare provider level was insufficient training and development of skills for providing healthcare to persons with amputations. Common barriers at the system level included inadequate funding of healthcare services for persons with amputations, and fragmentation of the healthcare, implying that each rehabilitation intervention is carried out in a different place. This fragmentation is worsened by deficient health information systems hindering the integration of various providers and the continuity of services. Preliminary findings from ongoing research that explored the accessibility of prostheses to 85 Colombian individuals with amputations [103] reported that while prostheses were prescribed to 15 of 85 patients (17.6%), only 3 patients received the prosthesis (3.5%). The average time between the date of amputation and the prosthesis prescription for the 15 patients was 147 days. The time between the date of amputation and receiving the prosthesis varied between 4 and 8 months.

#### Strategies for improving adherence to policy recommendations in low- and middle-income countries

At the patient level, strategies that positively impact patient experiences included establishing a structured program with clear goals and feedback on the rehabilitation process post-amputation. Telehealth, telerehabilitation and tele-education programs were also accepted by participants and improved their follow-up, increased their knowledge about their condition and improved health outcomes [60, 62, 72].

At the healthcare provider level, we identified a rate reduction of major amputations and diabetes-related lower limb ulcers by implementing specific healthcare arrangements and better pathways, and establishing special multidisciplinary teams [104]. One systematic review found that interactive telemedicine could reduce the cost of medical care, change the way care is organized, and improve accessibility to rehabilitation services. The strategies that showed a positive impact on provider experiences included the support of local health authorities and non-governmental organizations (NGOs) for the provision of educational resources to patients, education programs for clinicians [59, 61, 97] and using economic incentives for healthcare providers (97) (Table 3).

**Table 3:** Implementation strategies for improving adherence to policy recommendations in low- and middle-income countries

Level	Findings
Patient experience	• A high-quality systematic review (SR) from 2016 [71] evaluated the effects of goal setting and strategies such as planning and feedback on progress in adherence to health outcomes in adults with acquired disabilities participating in rehabilitation. The authors found that a structured approach to goal setting, compared with no goal setting, significantly increased health-related quality of life, emotional state and task-specific self-efficacy. However, no differences were found in the commitment of the individual patient with rehabilitation • The SR of Subedi et al., of low quality, evaluated the effectiveness of the implementation of cardiac telerehabilitation interventions. These interventions were highly accepted (71% and 99% of the participants) [60] • A high-quality SR from 2019 [62] examined the impact of interventions to improve the care of people with the cardiovascular disease living in rural areas. Among the interventions for cerebrovascular accident (CVA), those based on education provided improved patient knowledge and behavioural outcomes
Health outcomes	• A high-quality Cochrane SR, updated in 2015 to evaluate the effectiveness of interactive telemedicine (TM) [72] in patients with multiple conditions in which specialist care was required, showed that the use of TM has the potential to improve patient health outcomes. There is still uncertainty about the efficacy of TM in specific clinical situations and how it should be incorporated into the general health service. The data so far indicate, for example, that the use of TM in the treatment of heart failure seems to lead to similar health outcomes as in-person administration. There is also evidence that TM can improve blood glucose control in people with diabetes • A 2019 high-quality SR [62] examined the impact of interventions on improving cardiovascular disease care for people living in rural areas. Among the interventions for cerebrovascular accidents (CVAs), those based on the implementation of telemedicine improved the follow-up of survivors. Although the available evidence shows that these interventions can improve health processes, their impact on mortality and other important health outcomes has yet to be established • An SR with 57 studies by Meza-Torres [110], published in 2020, evaluated the impact of health services organizations on lower limb amputation rates in people with type 2 diabetes affected by foot ulcers. They identified that specific healthcare arrangements, better pathways of care, dedicated and multidisciplinary teams, and combined interventions could reduce the rate of major amputations in people with type 2 diabetes and diabetes-related lower limb ulcers
Costs	• A critically low-quality SR on using economic evidence to prioritize healthcare in low-income countries showed that most studies base their analyses on minimal efficacy and effectiveness data or the opinion of experts. In addition, extrapolation of the best available international evidence from high-income to low-income settings is often required [67] • A low-quality SR evaluated aspects of the implementation of cardiac telerehabilitation interventions. A 72–90% probability of cost-effectiveness was reported for a text message (SMS) intervention and a moderate-to-high likelihood that telerehabilitation would be more cost-effective than centre-based rehabilitation [60] • A high-quality Cochrane SR, updated in 2015, aimed to evaluate the effectiveness of interactive TM [72] in patients with multiple conditions in which specialist care was required. The review showed that the use of TM has the potential to reduce the cost of medical care, change the way care is organized and improve access
Provider experience	• In a critically low-quality SR, the authors identified facilitators and barriers to capacity building of the rehabilitation workforce in low- to middle-income countries (LMICs). Among the reported facilitators, it was found that some LMICs received help in the form of support in colleges, training programs, universities and collaborations with international NGOs. The support of the local government was present in various capacities, such as the provision of educational resources, international exchange programs and development of clinical and educational services at a distance [59] • A moderate-quality, institutional-level SR to improve the palliative care context’s organization evaluated some implementation strategies to convince healthcare providers to use new evidence [61]. The strategies that proved to be effective were education (conferences, study days, role-play sessions, interactive education, educational outreach visits and education facilitated by technological means); process mapping, feedback, and family and multidisciplinary meetings to improve communication and shared decision-making; and multifaceted implementation strategies • An overview of SRs, published in 2017 [58], provided a summary of the effects of service delivery arrangements for health systems in low-income countries. In general terms, the participation of different health actors such as nursing personnel, primary care physicians and specialists; care by levels of care; integration between hospital, outpatient and community care settings; and involvement of virtual, remote or technology-based care all seem to respond to the changing and growing needs of low-income countries with regard to healthcare. All included reviews reported that primary research is needed for at least one of these outcome categories: patient outcomes; access, coverage, or use; attention quality; or use of resources

^*^Findings drawn from systematic reviews

#### Strategies for comprehensive care and continuity of rehabilitation services for persons with amputations

At the patient level, strategies that positively impacted patient experiences included providing home-based training by community volunteers, which improved independence, mobility, communication skills and social integration [95]. Economic incentives also improved patients’ self-esteem and their status in the family and the community [95]. Regarding health outcomes, we only identified indirect evidence from a systematic review, showing that in people with acquired brain injury, multidisciplinary rehabilitation versus local services was associated with earlier functional improvements [69].

At the healthcare provider level, we found some interventions that positively impacted provider experiences; those interventions included planning rehabilitation services considering the organization of providers, type of health insurance and availability of resources. The healthcare delivery should consider the mode of service delivery (i.e. hospital-based, home-based or telerehabilitation); phases of care (habilitation, pre-habilitation, acute rehabilitation care, subacute rehabilitation care, post-acute rehabilitation care and long-term/chronic rehabilitation care); vertical (according to the needs of patients and across different levels of care) and horizontal (along the continuum of care) integration of the continuum of healthcare; and organization and competencies of multidisciplinary teams [105]. Other strategies identified were related to managing the healthcare service delivery on the basis of performance, measuring outcomes at all levels and promoting collaboration among different health sectors and healthcare providers [80] (Table 4).

**Table 4:** Implementation strategies for comprehensive healthcare and continuity of rehabilitation services for individuals with amputations

Level	Findings
Patient experience	• In a nonsystematic review, which examined 29 reports from 22 countries in Asia, Africa and Central America to assess the effectiveness of community-based rehabilitation interventions, it was found that the provision of in-home training by community volunteers resulted in greater independence, mobility, communication skills and social integration in at least 50% of the people. Additionally, the income of people with disabilities improved through economic interventions, and this improved their self-esteem and their status in the family and the community. However, it was much less clear whether people with disabilities can achieve financial independence as a result of these interventions [95] • A moderate-quality SR [63] that looked at the heterogeneous outcomes of people with spinal cord injury in terms of community integration found 10 common factors that affect rehabilitation outcomes to varying degrees. It describes them in three categories: The first is the factors related to the injury (cause, level and severity, age and duration). The second are personal factors (demographics, physical or mental illness and other comorbidities, personal abilities and attitude towards disability). The third is socioenvironmental factors (access to formal support, availability of informal support, geographic variation, other aspects of the environment)
Health outcomes	• In a high-quality 2012 SR [65], the authors evaluated the effectiveness of interventions (case management, shared care and interdisciplinary teams) aimed at improving continuity of care for patients with cancer, the provider of medical care and the results of the process. No significant differences were found in health-related outcomes between patients assigned to interventions and those assigned to usual care • In a high-quality Cochrane SR, the authors compared multidisciplinary rehabilitation versus commonly available local services or lower levels of intervention for people with acquired brain injury (ABI). They found that for participants with moderate-to-severe ABI who were already in rehabilitation, more intensive programs are associated with earlier functional improvements with strong evidence in favour, and with moderate evidence in favour, that continuous outpatient therapy could help maintain outcomes and achievements obtained in early post-acute rehabilitation. The context of multidisciplinary rehabilitation seems to influence the results. Specialized inpatient rehabilitation and specialized multidisciplinary community rehabilitation may provide additional functional benefits (weak evidence in favour) [69] • A 2011 moderate-quality SR [68] assessed whether the evidence supports coordinated transition of services in post-acute care for patients hospitalized with a first stroke (CVA) or recurrent myocardial infarction (MI). Moderate evidence was found to support the benefit of two components of hospital-initiated planning and early assisted discharge for stroke patients; this was associated with a reduction in length of hospital stay and referral for speciality follow-up after MI, which was associated with a reduction in mortality
Costs	• A moderate-quality scoping review on effectiveness and cost-effectiveness of prosthetic and orthopaedic interventions found that of 346 randomized controlled trials (RCTs), only 4 evaluated prosthetic interventions (all in lower limbs). The main research emphasis to date in this area has been to examine the effectiveness of specific prosthetic or orthopaedic interventions, without evaluating profitability. Only 5 of the 342 RCTs that examined the provision of braces examined the cost-effectiveness of the tested interventions. The authors note that a study published recently in a special issue of *Military Medicine* analysed Medicare claims data and found that people who received lower limb prostheses had comparable Medicare episode payments and better outcomes than those who did not receive prosthetics. People who were given prosthetics were more likely to receive extensive outpatient therapy, and this was associated with fewer hospitalizations, fewer emergency room visits and less care in institutions. This review concludes that information on cost-effective prosthetic/orthopaedic interventions is needed as this would provide an evidence base for their provision to healthcare providers and policy-makers [77]
Provider experience	• A 2017 moderate-quality scoping review [80] aimed to identify the relevant models and elements for integrated care in patients with multimorbidity. The authors reported that Wagner’s chronic care model (CCM) and guided care model (GCM) were the most frequently mentioned. Continuity of care was described as the key to the success of integrated care. One element commonly mentioned was the use of performance-based assessment/management or the measurement of care outcomes at all levels. Funding factors were the least frequently encountered. It was considered relevant to have an information exchange system or an interoperable system to exchange information between professionals, patients and informal caregivers and thus optimize the care process • An evidence brief [75] for the care of older adults in Ontario, Canada, evaluated specific programs and services to help older adults lead healthy and independent lives in their own homes, and showed that current health system arrangements do not support full continuity of care and present needs in financial, governance and delivery arrangements. The summary offers, on the basis of a review of the evidence, an approach to address the problem on the basis of three elements: o Empower older adults (OAs) and their families in ways that support healthy ageing, through strategies that improve self-management; develop evidence-based tools that provide guidance to OAs and their families about the care and services they need and how to access them; provide telehealth and e-health more generally; provide electronic medical records that are easily accessible by OAs and their family and friends to manage their information; and provide transportation programs and financial protections for older adults and their families o Coordinate integrated healthcare services that are based on the needs of OAs, guaranteeing and unifying access to multiple healthcare services and settings, and providing flexible home care supports and episodic and respite care that help older adults avoid care in health institutions o Provide personalized integrated healthcare service packages with the combination and number of services determined on the basis of the specific needs of each OA (determined through a coordinated evaluation mechanism), and use multidisciplinary teams or units that can evaluate and coordinate the provision of appropriate combinations of health services that meet the specific needs of OAs. Provide a process to assess consumer satisfaction and redesign services tailored to their needs • An evidence brief from 2013, also from Ontario (Canada), evaluated the growing problem of patients with multimorbidity (MM) [74], showing that the health system arrangements did not meet the care needs of this population. The authors raised three important elements for designing integrated approaches to support people with MM in Ontario: o Support primary care, community care and other providers to adapt and implement models of care for patients with MM that improve patient experience and health, and keep per capita costs manageable. Evidence shows that interventions targeting more specific changes in the provision of care within an organization (for example, coordinated integrated treatment programs) were more effective than those with a broad approach and that “complex and multifaceted pharmaceutical care” (such as pharmacist outreach interventions, automated medication alert detection and clinical pharmacist interventions in various settings) reduced inappropriate medication use and medication-related adverse events. o Allow primary care, community care and other providers to identify and use guidelines (or pathways of care) that meet the needs of patients with multimorbidity. The latest reviews found beneficial effects for educational materials, local opinion leaders, educational outreach/practice facilitation, auditing and feedback, computerized decision support and multifaceted interventions o Enable primary care, community care and other providers to efficiently support self-management of patients with multimorbidity. Two systematic reviews found benefits (e.g. better physical and mental health outcomes) for patient education and family interventions as possible approaches to help patients with MM use self-management resources. Several reviews described the benefits of information and communication technology, home support and a number of interventions aimed at supporting appropriate drug use by consumers • A moderate-quality scoping review found that the provision of rehabilitation services at different levels requires an adequate referral system; the establishment of a regional centre for rehabilitation; rehabilitation centres close to existing regional hospitals; consideration of a specific referral pathway to provide rehabilitation care equipment; and accurate, consistent and effective organizational training and management [76] • An SR from 2018, which evaluated the barriers, facilitators and routes for cardiac rehabilitation (CR) in people living in rural areas of high-income countries, raises some priorities to implement cardiac rehabilitation in these areas such as a systematic referral process, based on well-defined criteria, with individualization of CR. It is important that the programs are flexible to be able to adapt to different demographic profiles, health conditions, and geographic location (e.g. rural and remote areas). Alternative models such as home-based programs with telephone support, telehealth, specifically designed applications and community participation are facilitators [70] • An SR of critically low quality describes a conceptual framework of interprofessional or interorganizational collaboration in the health field. Among the structural and organizational characteristics important for interprofessional collaboration in institutions were the following: (1) Developing and promoting a shared vision of goals and a patient-centred orientation. (2) Implementing an evaluation of the interdisciplinary effort and reflection on the process. (3) Formalizing collaboration between organizations and clarifying the roles and responsibilities of each actor. (4) Creating formal channels of communication, as well as providing time and space for informal communication between individuals and groups. (5) Assigning adequate resource support in terms of time, space and human and material resources for the completion of tasks. (6) General planning of local institutional structures (67) • A nonsystematic review on prosthetic and orthotic service delivery in low-income countries evaluated current models of service delivery. There is a consensus in the literature that appropriate technology is required for low-income countries. However, there is no agreement on what appropriate technology means. The importation of orthopaedic and prosthetic technology from industrialized countries often does not meet the needs of people in low-income countries. Strategic planning, focused administration and effective management are required for this service provision. The economic viability of these services and the accessibility of these services are also important owing to socioeconomic problems, low literacy rates, language barriers and the lack of adequate transportation facilities [90]

^*^Findings drawn from reviews only; individual studies were not included

At the health system level, we found one review that showed that patients who received the prosthesis were more likely to receive extensive outpatient therapy. This was associated with fewer hospitalizations, fewer emergency room visits and less in-hospital healthcare.

## Discussion

In this study, we developed two evidence briefs to inform the implementation planning of recommendations on EF for persons with a STEMI and the provision of rehabilitation services for persons with amputations. Although both briefs were developed separately, we identified common facilitators and barriers for implementing these recommendations.

A common barrier identified in both briefs related to patient characteristics such as living in rural areas or low-socioeconomic-status communities [15, 16, 22, 24, 44, 48, 51, 53, 54, 56]. These factors negatively affected access to healthcare services, given that these communities are commonly underserved and face difficulties in transportation [15, 22, 53, 54, 56]. In Colombia, 19% of the population lives in rural areas [106], and almost 40% of the rural population lives in poverty [107], which means that implementation strategies focused on improving timely access to and continuity of services for the rural and poor population are essential in the Colombian context.

At the individual healthcare provider level, we found that the lack of training was related to the low implementation of EF for persons with a STEMI and the lack of rehabilitation services for persons with amputations [16, 20, 22, 24, 26, 42, 44, 47, 53, 56]. This is especially important given that in rural areas there is no availability of cardiologists, physiatrists or any other specialist. Therefore, the training of general doctors is fundamental to support the implementation of both recommendations as the country has enough human resources accessible for the entire population. This finding indicates that the education sector has an essential role in planning for implementing these recommendations [22, 42, 44].

At the health system level, we found that a lack of healthcare networks integrating community facilities and complex healthcare institutions, and the absence of healthcare pathways that facilitate navigating the health system and clearly understanding the functions of each stakeholder, contribute to the fragmentation in the provision of healthcare and represent an accessibility barrier [12, 14, 18, 22–24, 37, 42, 45, 53, 55]. This finding appears in a context where private intermediaries, private health insurers and private providers compete instead of collaborate, and the strong interests of this private sector constrict the stewardship of the health system.

Some of these findings are consistent with primary research conducted in Colombia and were also recognized in a stakeholder dialogue held in 2020 for the socialization of the amputee comprehensive healthcare pathway, with the participation of representatives from the CMoH, health insurance companies, healthcare providers, academics, clinicians from different areas and patients [32, 108].

We also identified common facilitators in both evidence briefs. For instance, at the institutional level, the areas in charge of quality monitoring and improvement could facilitate the implementation of CPG recommendations because they help identify suboptimal care sources within their institutions and prehospital providers. In the Colombian health system, healthcare institutions must have procedures for developing institutional CPGs and monitoring the implementation for their 10 most prevalent conditions [22, 49, 53, 109].

### Implications for practice

When analysing strategies for implementation, one relevant approach identified in both briefs is to create healthcare networks with clear pathways that facilitate continuity and integration of healthcare services between the rural and urban areas, as well as among different healthcare providers (i.e. prehospital, community facilities and hospitals). For the implementation of EF, CCNs could be especially beneficial for the population living in rural areas, far away from coronary units, with difficulties in transportation, or who might face delays in access to reperfusion treatments. All these situations are highly likely among the Colombian population. STEMI care pathways in Colombia might help integrate prehospital care, community healthcare facilities and hospital care to provide timely access to reperfusion. Timely fibrinolysis gives a 24-h window to the referral to a PCI centre where PPCI or pharmaco-invasive therapy is available. Evidence about the effectiveness of EF and its positive impact on reducing morbidity and mortality after STEMI is overwhelming. It indicates that Colombia is missing implementing an intervention that might save lives and resources in the health system [12, 16]. Implementing early fibrinolysis for persons with a STEMI requires the commitment of scientific medical organizations, policy-makers, health insurance companies, healthcare providers, universities and citizens [19, 24], as well as encouraging community engagement in first aid training as part of the so-called chain of survival for patients with STEMI. As the authors of this brief, we consider it essential to promote the establishment of a coalition of stakeholders that advocate for the implementation of early fibrinolysis in Colombia.

Implementing healthcare networks with clear and comprehensive pathways is also paramount to guarantee the continuity and integration of healthcare rehabilitation services for persons with amputations. These pathways should consider implementing information and communication technologies such as telemedicine, telerehabilitation and tele-education, which could be beneficial for patients living in rural areas with difficulties accessing appropriate rehabilitation institutions. Other chronic conditions showed that telerehabilitation is not inferior to in-person rehabilitation processes [104]. In addition, it is essential to implement educational strategies for patients and healthcare providers to improve the knowledge of the condition and the skills necessary to take care of the prosthesis and to achieve a satisfactory adaptation process. Individuals with amputations must access a comprehensive rehabilitation process that includes psychological care delivered close to their workplace or residency.

Concerning the healthcare systems arrangements, it is crucial to develop adequate information systems that allow the monitoring and measurement of meaningful outcomes for patients. Implementing comprehensive healthcare pathways must also promote that financial agreements between insurance companies and healthcare providers guarantee the provision of integral rehabilitation and not only specific rehabilitation activities. This will avoid fragmentation of services, such as the current practice where insurance companies contract one rehabilitation service (i.e. physical therapy) in one institution and another service (i.e. physiological therapy) in a different one.

### Strengths and limitations

The limitations of this study are related to the methodology of evidence briefs, which have less rigorous methods than a formal systematic review and include different types of studies and publications. For instance, we did not perform the inclusion assessment and data extraction in duplicate and by independent reviewers. However, these evidence briefs are not oriented to determine the effectiveness of interventions but to inform a further implementation planning process. The strengths of these briefs are that (1) these documents are easy for Colombian stakeholders to understand and consult, with the evidence of barriers, facilitators and strategies for implementing both CPGs; (2) the evidence analysis supports public policies; and (3) the identification of limitations in Colombian health systems for the implementation of the CPGs’ recommendations allows recognizing mechanisms to overpass these.

For the brief on persons with amputations, the main limitation is not having data regarding implementation of recommendations in Colombia. At this time, an ongoing project is aiming to evaluate the effectiveness of a strategy on the basis of telehealth to improve the implementation of the CPGs for persons with amputations. We hope to be able to analyse the causes of poor adherence to rehabilitation recommendations, prescriptions and prosthesis adaptation. Another limitation was the few studies with direct evidence to respond to the two systematic review questions: adherence to policy recommendations in low- and middle-income countries and how to maintain comprehensiveness and continuity of services for persons with amputations.

## Conclusions

We concluded that evidence briefs are an important tool to inform the prioritization of recommendations to be implemented. The evidence identified for each topic addressed in this study exposes the challenges for future implementation processes.

For EF in individuals with a STEMI, the barriers and facilitators identified by other countries are consistent with those recognized in our country. There is a lack of knowledge for recognizing ACS symptoms and management in patients and the general population, which delays seeking appropriate care. In healthcare providers, the uncertainties regarding the appropriate diagnosis and the proper treatment, which are more significant among general practitioners in rural areas, hinder the correct approach to an early reperfusion. Finally, health system fragmentation needs to be overcome with strategies such as CCNs, because it is one of the main barriers to access timely treatment.

For persons with amputations, our findings are consistent with primary research conducted in Colombia. For example, in one qualitative study about perceived barriers and facilitators related to the implementation of the guideline for the care of persons with amputations in Colombia, the authors identified individual barriers, such as the lack of knowledge and skills by health workers, and health system level barriers, such as the lack of integration networks between health institutions of different levels of complexity. The lack of integrated networks of care in the Colombian health systems is caused or promoted by the type of financial arrangements between insurance companies and healthcare institutions. In the dialogue, it was also recognized that the insurance companies must recognize their responsibility in the care, rehabilitation and risk management of their insured population, including extramural actions that allow for the decentralization of rehabilitation services.

## Data Availability

All data generated or analysed during this study are included in this published article and its supplementary information files.

## Abbreviations

ACS: Acute coronary syndrome
AMI: Acute myocardial infarction
CCN: Coronary care network
CMoH: Colombian Ministry of Health
CPG: Clinical practice guideline
EF: Early fibrinolysis
PPCI: Primary percutaneous coronary intervention
STEMI: ST-elevation myocardial infarction
